# Supplementary material for: Micrococcin P1 and P2 from Epibiotic Bacteria Associated with Isolates of Moorea producens from Kenya
Source: Mar Drugs. 2022 Feb 7;20(2):128. doi: 10.3390/md20020128 (PMC8878052; doi:10.3390/md20020128)

SUPPORTING INFORMATION (SI)

SI Figure S1 Denaturation of *Moorea producens* DNA

$CuSO_4.5H_2O$ assisted differential gDNA isolation of *Moorea producens*

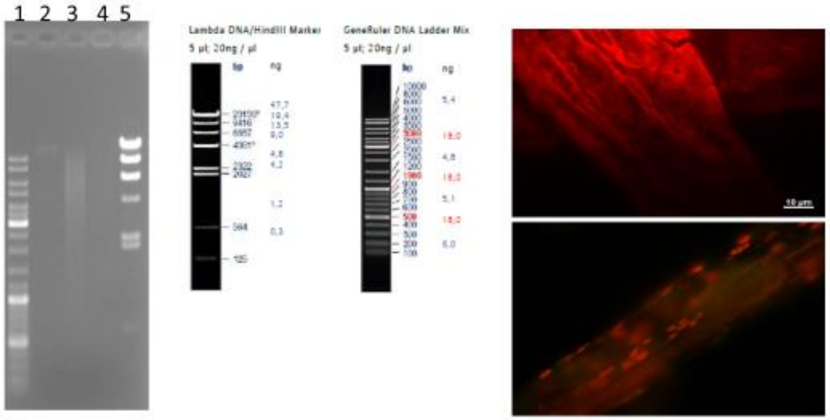

Lane 1: Gene ruler DNA ladder mix; **Lane 2:** *M. producens* treated with $CuSO_4.5H_2O$ for 30 min**; Lane 3 and Lane 4** *M. producens* treated with $CuSO_4.5H_2O$ for 90 min and 180 min respectively; **Lane 5:** Lambda DNA/Hind III marker. Top right – image of *M. producens* filament treated with $CuSO_4.5H_2O$ for 30 min. Bottom right – image of a dying *M. producens* filament. Photos taken by the author using a Leica DMIRB inverted microscope

SI Figure S2. **Blasted sequences**

#1:1-879_Kenyan_*Moorea_producens*_Rep1

GGGAACCCTGACCCAGCGACGCCSCGTGAGGGAAGACGGCCTYCGGGTTGTAAACCTCTT

TTGTATGGGAAGAAGGATGTGACGGTACCATACGAATAAGTCCCGGCTAACTACGTGCCA

GCAGCCGCGGTAATACGTAGGGGACGAGCGTTGTCCGGAATTACTGGGCGTAAAGGGCGC

GCAGGCGGTTTGTCAAGTCAGCTGTTAAAAATGGGGGCTTAACCCTCATA-TGCTGTTGA

AACTGGCAGACTGGAGTGCTGTAGAGGCAAGTGGAATTCCCAGTGTAGCGGTGAAATGCG

TAGATATTGGGAAGAACACCGGTGGCGAAGGCGACTTGCTGGGCAGCAACTGACGCTGAG

GC

#1:1-636_Kenyan_*Moorea_producens*_Rep_2

GGGAACCCTGACCCAGCGACGCCGCGTGAGGGAAGACGGCCTTCGGGTTGTAAACCTCTT

TTGTWTGGGAAGAAGGATGTGACGGTACCATACGAATAAGTCCCGGCTAACTACGTGCCA

GCRGCCGCGGTAATACGTAGGGGACGAGCGTTGTCCGGAATTACTGGGCGTAAAGGGCGC

GCAGGCGGTTTGTCAAGTCAGCTGTTAAAAATGGGGGCTTAACCCTCATA-TGCTGTTGA

AACTGGCAGACTGGAGTGCTGTAGAGGCAAGTGGAATTCCCAGTGTAGCGGTGAAATGCG

TAGATATTGGGAAGAACACCGGTGGCGAAGGCGACTTGCTGGGCAGCAACTGACGCTGAG

GC

#1:1-634_Kenyan_*Moorea_producens*_Rep_3

GGGAACCCTGACCCAGCGACGCCGCGTGAGGGAAGACGGCCTTCGGGTTGTAAACCTCTT

TTGTATGGGAAGAAGGATGTGACGGTACCATACGAATAAGTCCCGGCTAACTACGTGCCA

GCAGCCGCGGTAATACGTAGGGGACGAGCGTTGTCCGGAATTACTGGGCGTAAAGGGCGC

GCAGGCGGTTTGTCAAGTCAGCTGTTAAAAATGGGGGCTTAACCCTCATA-TGCTGTTGA

AACTGGCAGACTGGAGTGCTGTAGAGGCAAGTGGAATTCCCAGTGTAGCGGTGAAATGCG

TAGATATTGGGAAGAACACCGGTGGCGAAGGCGACTTGCTGGGCAGCAACTGACGCTGAG

GC

#NR 102462.1:1-1479_*Cylindrospermum_stagnale*_16S_rRNA,_partial_sequence

CGAAAGCCTGACGGAGCAATACCGCGTGAGGGAGGAAGGCTCTTGGGTTGTAAACCTCTT

TTCTCAGGGAATAAA-AAATGAAGGTACCTGAGGAATAAGCATCGGCTAACTCCGTGCCA

GCAGCCGCGGTAATACGGAGGATGCAAGCGTTATCCGGAATGATTGGGCGTAAAGCGTCC

GCAGGTGGCTATGTAAGTCTGCTGTTAAAGAGCAAGGCTCAACCTTGTAAAGGCAGTGGA

AACTACATAGCTAGAGTGCGTTCGGGGCAGAGGGAATTCCTGGTGTAGCGGTGAAATGCG

TAGATATCAGGAAGAACACCGGTGGCGAAAGCGCTCTGCTAGGCCGCAACTGACACTGAG

GG

#JX912466.1:1-889_*Halospirulina_sp*._EF17(2012)_16S_rRNA_gene,_partial_sequence

CGAAAGCCTGACGGAGCAATGCCGCGTGGAGGTAGAAGGCCCACGGGTCGTGAACTTCTT

TTCCCGGAGAAGAAGCAA-TGACGGTATCTGGGGAATAAGCATCGGCTAACTCTGTGCCA

GCAGCCGCGGTAATACAGAGGATGCAAGCGTTATCCGGAATGATTGGGCGTAAAGCGTCT

GTAGGTGGCTTTTTAAGTCCGCCGTCAAATCCCAGGGCTCAACCCTGGACAGGCGGTGGA

AACTACCAAGCTGGAGTACGGTAGGGGCAGAGGGAATTTCCGGTGGAGCGGTGAAATGCG

TAGAGATCGGAAAGAACACCAACGGCGAAAGCACTCTGCTGGGCCGACACTGACACTGAG

AG

#AF510976.1:1-604_*Lyngbya_majuscula*_CCAP_1446/4_Rep_1

CGCAAGCCTGACGGAGCAAGACCGCGTGGGGGAGGAAGGCTCTTGGGTTGTAAACCCCTT

TTCTCAGGGAAGAACCAAATGACGGTACCTGAGGAATCAGCCTCGGCTAACTCCGTGCCA

GCAGCCGCGGTAATACGGAGGAGGCAAGCGTTATCCGGAATGATTGGGCGTAAAGCGTTC

GTAGGTGGCTATTCAAGTCTGCTGTCAAAGACAGAAGCTCAACTTCCGAAAGGCAGTGGA

AACTGAGTAGCTGGAGTGCGGTAGGGGCAGAGGGAATTCCCGGTGTAGCGGTGAAATGCG

TAGAGATCGGGAAGAACACCGGTGGCGAAAGCGCTCTGCTGGGCCGCAACTGACACTGAG

GG

#AF510967.1:1-604_*Lyngbya_majuscula*_CCAP_1446/4_Rep_2

CGCAAGCCTGACGGAGCAAGACCGCGTGGGGGAGGAAGGCTCTTGGGTTGTAAACCCCTT

TTCTCAGGGAAGAACCAAATGACGGTACCTGAGGAATCAGCCTCGGCTAACTCCGTGCCA

GCAGCCGCGGTAATACGGAGGAGGCAAGCGTTATCCGGAATGATTGGGCGTAAAGCGTTC

GTAGGTGGCTATTCAAGTCTGCTGTCAAAGACAGAAGCTCAACTTCTGAAAGGCAGTGGA

AACTGAGTAGCTGGAGTGCGGTAGGGGCAGAGGGAATTCCCGGTGTAGCGGTAAAATGCG

TAGAGATCGGGAAGAACACCGGTGGCGAAAGCGCTCTGCTGGGCCGCAACTGACACTGAG

GG

#AF368300.1:1-585_*Lyngbya_majuscula*

CGCAAGCCTGACGGAGCAAGACCGCGTGGGGGAGGAAGGCTCTTGGGTTGTAAACCCCTT

TTCTCAGGGAAGAACCAAATGACGGTACCTGAGGAATCAGCCTCGGCTAACTCCGTGCCA

GCAGCCGCGGTAATACGGAGGAGGCAAGCGTTATCCGGAATGATTGGGCGTAAAGCGTTC

GTAGGTGGCTATTCAAGTCTGCTGTCAAAGACAGAAGCTCAACTTCTGAAAGGCAGTGGA

AACTGAGTAGCTGGAGTGCGGTAGGGGCAGAGGGAATTCCCGGTGTAGCGGTGAAATGCG

TAGAGATCGGGAAGAACACCGGTGGCGAAAGCGCTCTGCTGGGCCGCAACTGACACTGAG

GG

#JX912376.1:1-854_*Planktothricoides_sp.*_EA12(2012)

CGAAAGCCTGACGGAGCAATGCCGCGTGGAGGTAGAAGGCCCACGGGTCGTGAACTTCTT

TTCCCGGAGAAGAAGCAA-TGACGGTATCTGGGGAATAAGCATCGGCTAACTCTGTGCCA

GCAGCCGCGGTAATACAGAGGATGCAAGCGTTATCCGGAATGATTGGGCGTAAAGCGTCT

ATAGGTGGCTTTTTAAGTCCGCCGTCAAATCCCAGGGCTCAACCCTGGACAGGCGGTGGA

AACTACCAAGCTGGAGTACGGTAGGGGCAGAGGGAATTTCCGGTGGAGCGGTGAGATGCG

TAGAGATCGGAAAGAACACCAACGGCGAAAGCACTCTGCTGGGCCGACACTGACACTGAG

AG

#EF613820.1:1-803_Uncultured_cyanobacterium_clone_GA94_16S_rRNA_gene

CGCAAGCCTGACCCAGCAACGCCGCGTGAGGGAAGAAGGTCTTCGGATTGTAAACCTCTG

TCCTTGGGGACGAAAAAAATGACGGTACCCAAGGAGGAAGCTCCGGCTAACTACGTGCCA

GCAGCCGCGGTAATACGTAGGGAGCGAGCGTTGTCCGGAATTACTGGGCGTAAAGGGTGC

GTAGGCGGCCTAGCAAGTCAGATGTGAAATCCCCGGGCTTAACTCGGGGGGTGCATTTGA

AACTGCTTGGCTTGAGTGCAGGAGAGGAAAGCGGAATTCCTAGTGTAGCGGTGAAATGCG

TAGATATTAGGAAGAACACCAGTGGCGAAGGCGGCTTTCTGGACTGCAACTGACGCTGAG

GC

#HE578055.1:1-1094_Uncultured_Oscillatoriales_cyanobacterium_partial_16S_rRNA_gene,_clone_TPB_GMAT_CYANO_26

CGAAAGCCTGACGGAGCAATGCCGCGTGGAGGTAGAAGGCCCACGGGTCGTGAACTTCTT

TTCCCGGAGAAGAAGCAA-TGACGGTATCTGGGGAATAAGCATCGGCTAACTCTGTGCCA

GCAGCCGCGGTAATACAGAGGATGCAAGCGTTATCCGGAATGATTGGGCGTAAAGCGTCT

GTAGGTGGCTTTTTAAGTCCGCCGTCAAATCCCAGGGCTCAACCCTGGACAGGCGGTGGA

AACTACCAAGCTGGAGTACGGTAGGGGCAGAGGGAATTTCCGGTGGAGCGGTGAAATGCG

TAGAGATCGGAAAGAACACCAACGGCGAAAGCACTCTGCTGGGCCGACACTGACACTGAG

AG

#HE979756.1:1-598_Uncultured_Oscillatoriales_cyanobacterium_partial_16S_rRNA_gene,_DGGE_band_CYA28

CGAAAGCCTGACGGAGCAAGACCGCGTGAGGGAAGACGGCCTATGGGTTGTAAACCTCTT

TTGATAGGGAAGAAG-GACTGACGGTACCTATCGAATCAGCCTCGGCTAACTCCGTGCCA

GCAGCCGCGGTAATACGGAGGAGGCAAGCGTTATCCGGAATTATTGGGCGTAAAGCGTCC

GTAGGTGGTTGGTCAAGTCAGCTGTTAAAGCGCGGAGCTTAACTCCGTAACGGCAGTGGA

AACTGGTCAGCTAGAGTGCGATAGGGGCAAGGGGAATTCCCAGTGTAGCGGTGAAATGCG

TAGATATTGGGAAGAACACCGGTGGCGAAAGCGCCTTGCTGGGCCTGCACTGACACTGAG

GG

SI Figure S3 Micrococcin P1 and P2 chromatogram (MS and UV at 220 nm)

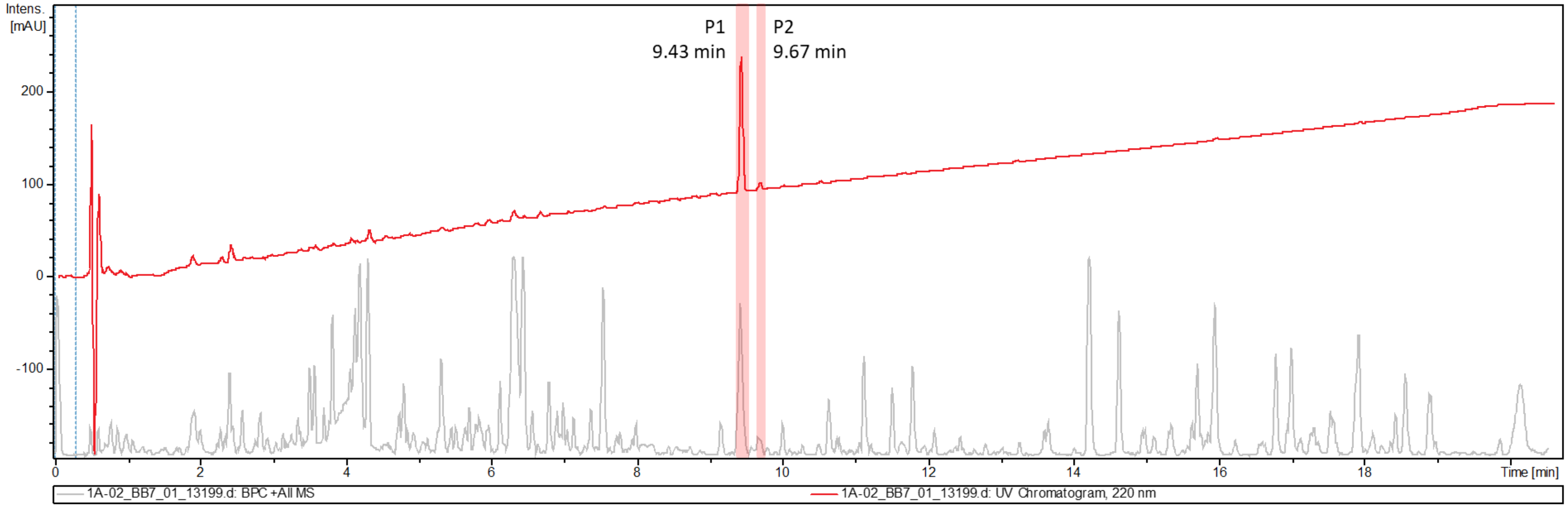

SI Figure S4 **Micrococcin P1 MS/MS spectrum**

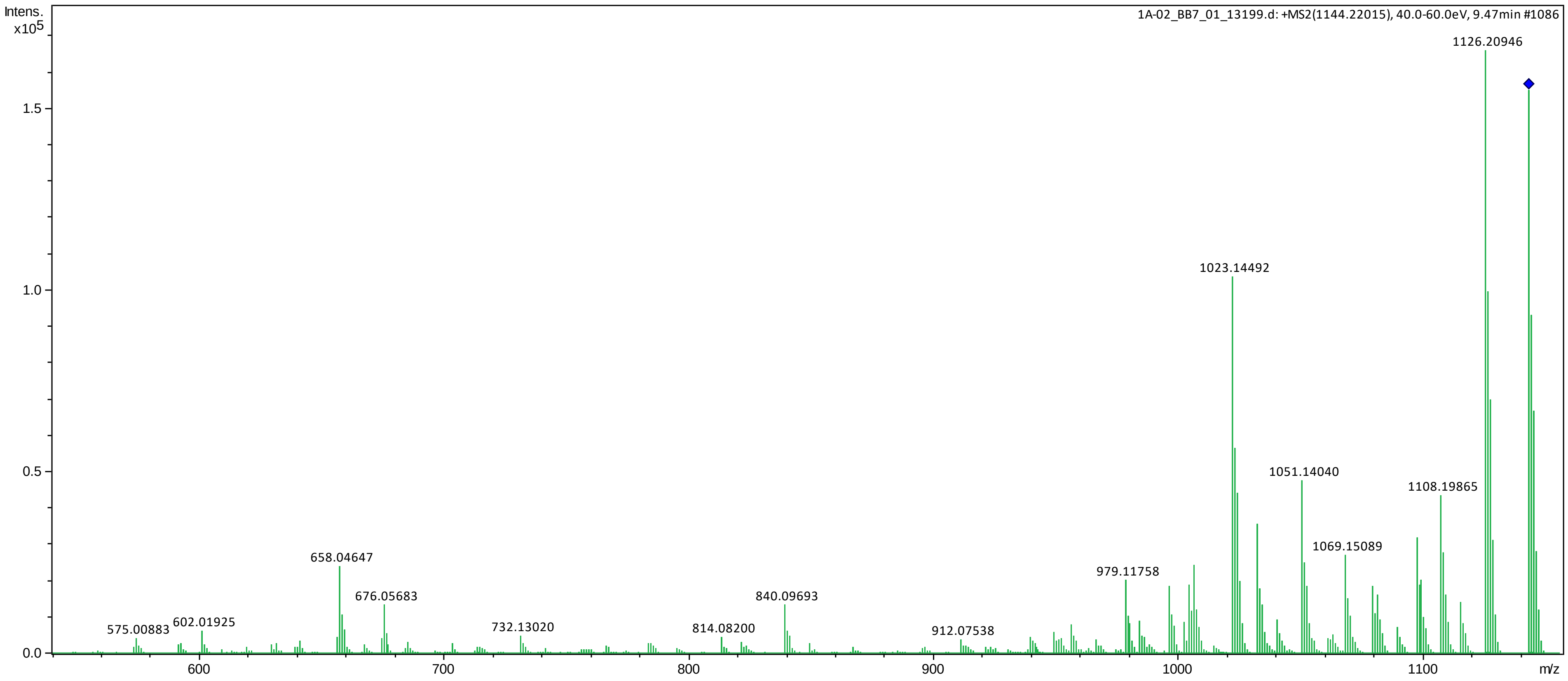

SI Figure S5 **Micrococcin P1 annotated MS/MS spectrum**

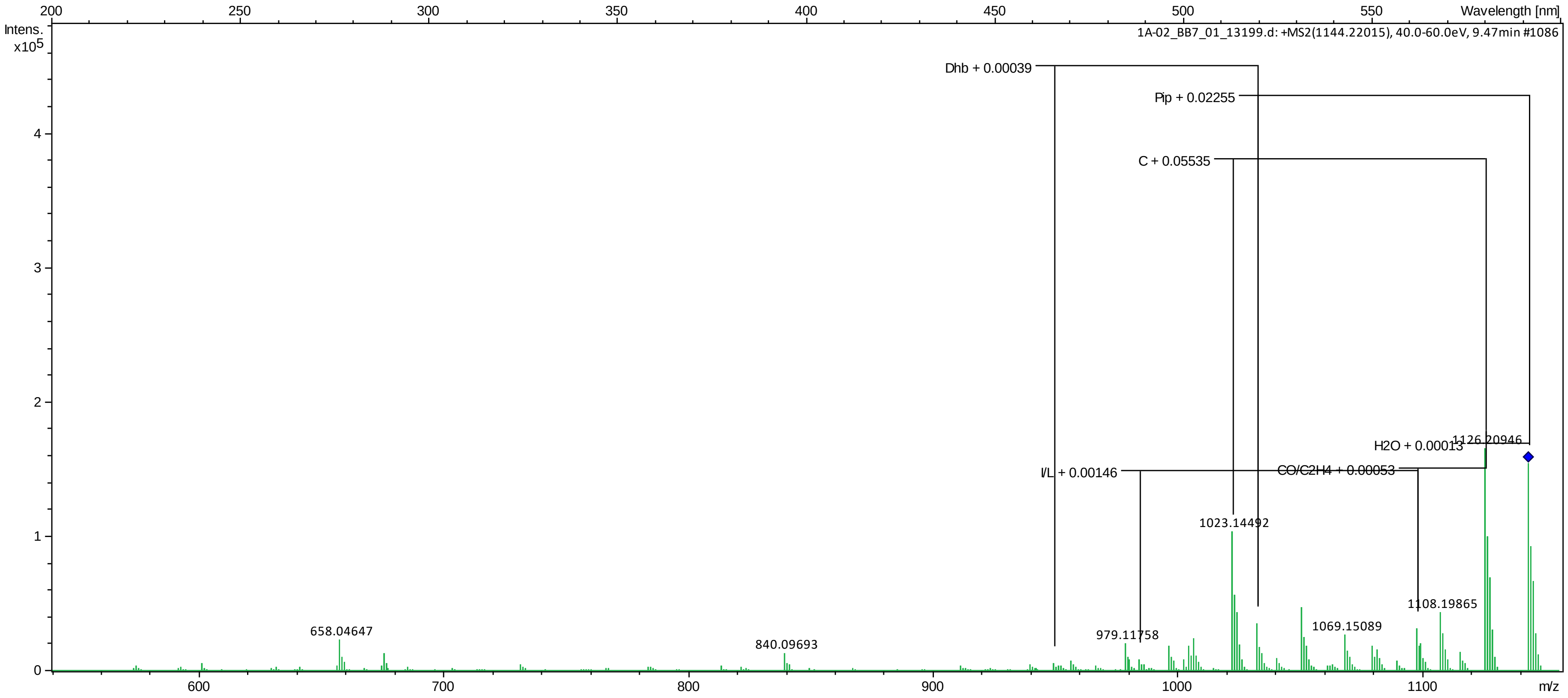

SI Figure S6 Micrococcin P2 MS/MS spectrum

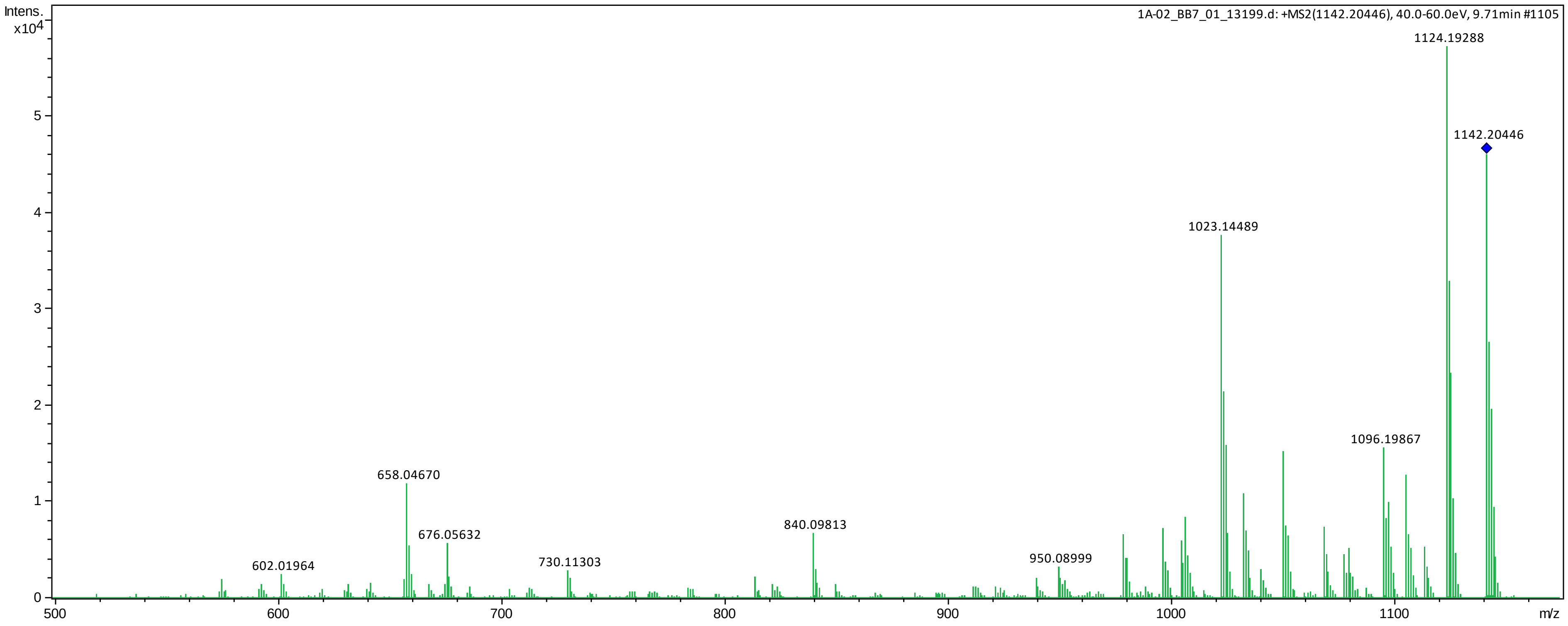

SI Figure S7 Micrococcin P2 annotated MS/MS spectrum

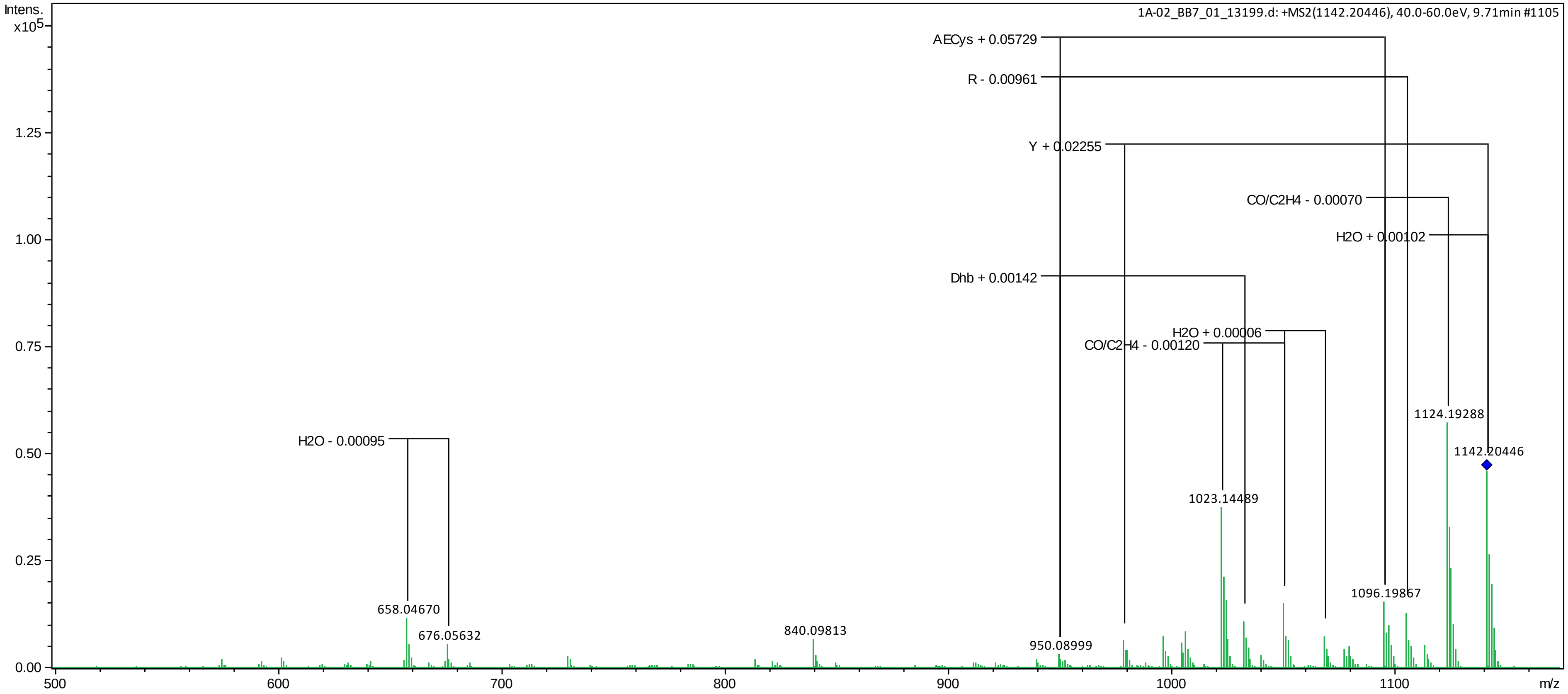

SI Figure S8 Activity of micrococcin P1 against *S. aureus* Newman

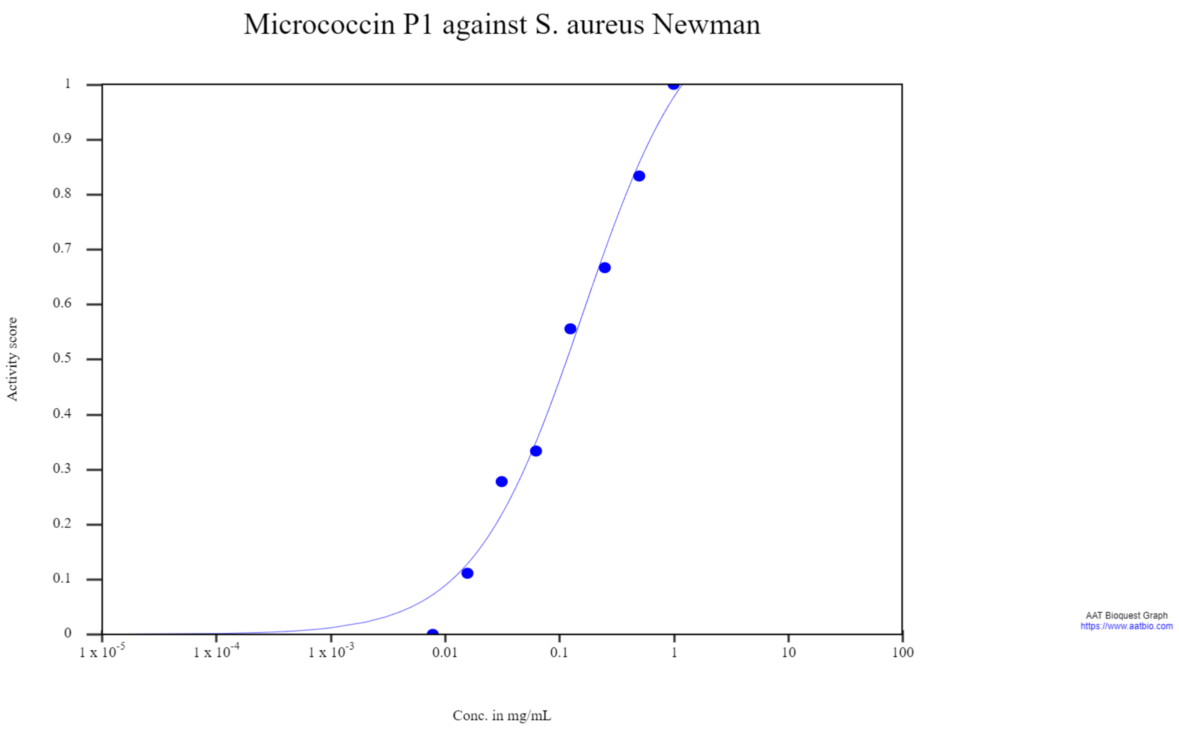

Supplement: Supplementary file 1 [file marinedrugs-20-00128-s001.zip › marinedrugs-1312685-supplementary.pdf]
